# Changes in the Chemical Composition and Decay Resistance of Thermally-Modified *Hevea brasiliensis* Wood

**DOI:** 10.1371/journal.pone.0151353

**Published:** 2016-03-17

**Authors:** Elias Taylor Durgante Severo, Fred Willians Calonego, Cláudio Angeli Sansígolo, Brian Bond

**Affiliations:** 1 Department of Forest Science, Faculty of Agricultural Sciences, São Paulo State University-UNESP, Botucatu, São Paulo, Brazil; 2 Department of Sustainable Biomaterials, Brooks Forest Products Center, Virginia Tech, Blacksburg, Virginia, United States of America; USDA Forest Service, UNITED STATES

## Abstract

In this study the effect of thermal treatment on the equilibrium moisture content, chemical composition and biological resistance to decay fungi of juvenile and mature *Hevea brasiliensis* wood (rubber wood) was evaluated. Samples were taken from a 53-year-old rubber wood plantation located in Tabapuã, Sao Paulo, Brazil. The samples were thermally-modified at 180°C, 200°C and 220°C. Results indicate that the thermal modification caused: (1) a significant increase in the extractive content and proportional increase in the lignin content at 220°C; (2) a significant decrease in the equilibrium moisture content, holocelluloses, arabinose, galactose and xylose content, but no change in glucose content; and (3) a significant increase in wood decay resistance against both *Pycnoporus sanguineus* (L.) Murrill and *Gloeophyllum trabeum* (Pers.) Murrill decay fungi. The greatest decay resistance was achieved from treatment at 220°C which resulted in a change in wood decay resistance class from moderately resistant to resistant. Finally, this study also demonstrated that the influence of thermal treatment in mature wood was lower than in juvenile wood.

## Introduction

*Hevea brasiliensis* (rubber wood) is a tree native to Brazil and is planted in many countries for the production of latex. The tapping of rubber trees starts between the fifth to seventh year after planting and then continues for 25 to 30 years. After 30 years a decline in latex production makes further tapping of the trees uneconomical. In the past, felled rubber trees were either burned on the spot or used as fuel for steam engines, brick making or latex curing. Its favorable qualities and light color make a good substitute for many species. Rubber wood is now one of the major resources for making furniture for export and for the production of panel products, such as particleboard, fiberboard (MDF), wood fiber cement-bonded particleboard, and plywood.

Biological durability is one of the most important properties of wood and assessment of durability is of great importance in appropriate material selection; both to avoid unnecessary spending caused by the need to replace damaged parts and to reduce the impacts on remaining natural forests [1, 2]. The high carbohydrate content (sugars and starches) stored in parenchyma cells and absence of extractives is crucial to *H*. *brasiliensis* susceptibility, and it has been the main reason why this species become less attractive for wood processing industries and has almost been neglected in the past [1–3]. While the woods biological durability can be increased by impregnation with chemical products, a negative side-effect is that the substances often have adverse health and environmental properties. Many studies have demonstrated that heat treatment can improve wood biological durability [4–6]. The low affinity of heat-treated wood to water and the availability of food (hemicelluloses) to fungi are reduced; new molecules that act as fungicides are produced, making the recognition of the substrate by fungi difficult. Thus, increasing the biological durability of wood by thermal modification is considered to be more acceptable [7].

Thermal treatments exposing wood to temperatures approaching 200°C for several hours can change its chemical composition. Severo et al [8] showed that thermally treating the juvenile and mature wood of *Pinus elliottii var*. *elliottii* at 200°C resulted in a significant increase in the extractives, the insoluble, soluble, and total lignin contents, and a significant reduction in the holocellulose content. Brito et al [9] showed that thermal treatment of *Pinus caribaea var*. *hondurensis* at 180°C caused a decrease in the arabinose, galactose, mannose, and xylose contents. Esteves et al [10] demonstrated that the degradation of lignin and hemicelluloses caused an increased in extractive content with thermal modification of eucalypt wood.

Heating of wood to high temperatures also influences its response to dimensional changes caused by changes in relative humidity due degradation of the hemicelluloses and the amorphous region of cellulose, thereby contributing to the increase in the degree of crystallinity. In addition, a cross-linkage between the lignin and the polymers occurs owing to the thermal degradation of the wood and are responsible for the decrease in the hygroscopicity, and an improvement of their dimensional stability [4, 5, 11–16].

There is no information about the chemical changes, effects on equilibrium moisture content (EMC), and impact on biological resistance in the literature regarding the thermal-modification of *H*. *brasiliensis* solid wood. Thus, the aim of this study was to evaluate the effects of thermal treatment on chemical composition, the changes in EMC and biological resistance to decay fungi of juvenile and mature wood from *Hevea brasiliensis* (Rubber wood).

## Materials and Methods

Wood from 53-year-old ungrafted *Hevea brasiliensis* trees from an area located in Tabapuã (20° 57’ 50” South Latitude and 49° 01’55” West longitude), São Paulo State, Brazil was used in this study. Five trees were felled and sectioned into 3.3 m logs. Rubber trees, indigenous to the Amazon Vally of South America, were introduced to São Paulo State as reforestation, so no specific permissions were required for logging activity. Moreover, this study did not involve endangered or protected species.

The logs with diameters between 35 and 40 cm were then cut into flat sawn boards. Boards containing the pith were cut into 34 mm thick pieces for this study. Subsequently, all the boards were kiln-dried up to 10.0% moisture content. The five dried boards were planed to 32 mm thickness and cut into smaller pieces measuring 0.60 m in length. Regions with cracks and knots were discarded. One piece was kept in its original condition (untreated), and the other pieces were thermally treated.

### Thermal treatments

The material was placed in a modified electric oven with a programmable controller. The treatment started at an initial temperature of 100 over a period of 14 h and then was increased (1.34°C/minutes) up to 180°C, to 200°C and to 220°C and maintained over a period of 2.5 h according to the application of the patent developed by Severo and Calonego [17]. After the thermal treatment, the oven was turned off, and the wood pieces were allowed to cool inside until they reached 30°C. Specimens were cut from all the boards (untreated and thermally modified woods) to test for chemical characterization, EMC and decay resistance for both juvenile and mature wood. The juvenile and mature wood regions were defined according to Ferreira et al [18].

### Chemical properties

Samples of untreated and thermally modified mature and juvenile wood were transformed into chips and then milled. The material used for chemical analysis was classified between 40 and 60 mesh. The extractives content was determined by extractions sequences with ethanol/toluene 1/2 (v/v), ethanol and hot water [19]. The acid-insoluble Klason lignin [20], acid-soluble Klason lignin [21], and holocellulose contents [holocellulose = 100 − (% Lignin + % Extractives Totals)] were determined in extractive-free wood. The sugars (arabinose, galactose, xylose, manose and glucose) were analyzed by High-Performance Anion-Exchange Chromatography with Pulsed Amperometric Detection (HPAE-PAD) in the filtrate obtained of the acid-insoluble Klason lignin. The analysis was carried out by using a chromatograph Thermo-Dionex, ICS-5000 with Pulsed Amperometric Detection, CarboPac PA 1 column and NaOH 0,5M as eluent a flow rate of 1 mL/min. [22].

### Accelerated laboratory tests of decay resistance

ASTM D-2017 [23] was used to evaluate the effect of thermal treatment on the biological durability of *H*. *brasiliensis* wood. Samples tested were cut approximately 40 mm from the pith and 40 mm from the bark and measured 25 by 25 by 9 mm (tangential, radial and longitudinal direction, respectively). Samples were obtained according to ASTM D-2017 [23]. Although this standard requires only six samples, we used fifteen obtained from five boards to characterize each of the treatments (untreated and thermally modified at180°C, 200°C and 220°C for both juvenile and mature wood for a total of 240 samples).

The wood samples were dried at 103±2°C, until they were of a constant weight. As recommended by ASTM D-1413 [24], the initial oven-dry weight (WI) of each test block was determined. After oven-dry weight, the test blocks were placed in the culture bottles, with the cross-section face down on the feeder strip. The cultures of *P*. *sanguineus* (collected from mycology collection of the Laboratory of Forest Pathology from Dept. of Vegetal Production of FCA-UNESP, Botucatu, SP, Brazil) and *G*. *trabeum* (collected from mycology collection of the INPA, Manaus, AM, Brazil and identified by ID 408) fungi were incubated in an incubation chamber in the dark to promote the growth of the fungus at 26.7 ± 1°C and 70 ± 4% relative humidity for 12 weeks. The percent weight loss in the individual test blocks was then calculated to provide a measure of the relative decay susceptibility or, inversely, the decay resistance of the untreated and thermally-modified *H*. *brasiliensis* wood.

### Statistical analysis

For the evaluation of EMC, chemical composition and biological resistance to decay fungi a Kolmogorov-Smirnov’s normality test at 5% significance was performed. All variables had normal distribution, except galactose content and weigh loss by the fungus *G*. *trabeum*. The normalization of the variable weight loss by the fungus *G*. *trabeum* was performed raising it to 0.0732 (Y^0.0732) according recommended by conversion of Box Cox.

Subsequently, a parametric test two-way (ANOVA) at 5% significance was performed taking into account the type of wood and the thermal treatment, as well as Tukey’s test at 5% significance for the comparison of the means in EMC, extractive, lignin, holocellulose, arabinose, xylose, mannose and glucose contents and weight loss of wood exposure to fungus *P*. *sanguineus* and *G*. *trabeum*.

For galactose content an extension of non-parametric Kruskal Wallis test (Scheirer-Ray-Hare) was performed taking into account the type of wood and the thermal treatment, showing there was only the influence of temperature of thermal treatment. The statistical software used was the Jandel SigmaStat version 2.0.

## Results and Discussion

### Chemical properties of thermally modified wood

The p value of Kolmogorov-Smirnov’s normality test for extractive, lignin, holocellulose, arabinose, galactose, xylose, mannose and glucose contents were 0.302, 0.582, 0.132, 0.070, 0.003, 0.055, 1.000, and 0.493, respectively. The extractive, lignin and holocellulose contents of juvenile wood from untreated *H*. *brasiliensis* were 5.96%, 19.25%, and 73.55%, respectively (Table 1) and in mature wood, were 5.83%, 18.18%, and 73.89%, respectively. These results are similar to those cited by Severo et al [25].

**Table 1:** Chemical properties of juvenile and mature woods of *Hevea brasiliensis* thermally modified at various temperatures.

Properties	Treatment temperature			
	Untreated	180°C	200°C	220°C
Extractives (%)				
*JW*, *C*.*V*.	13.0	32.7	20.0	18.7
*JW*: *Avg*.	5.96 a	8.51 b	10.86 b	11.84 b
*JW*, *↑ (%)*		42.8	82.2	98.7
*MW*, *C*.*V*.	2.9	38.0	19.4	15.8
*MW*, *Avg*.	5.83 a	8.24 b	8.06 b	9.55 b
*MW*, *↑ (%)*		41.3	38.3	63.8
*↓ (%)*	-2.2 *	-3.2 *	-25.8 *	-19.3 *
Klason L. (%)				
*JW*, *C*.*V*.	10.0	7.9	7.2	22.1
*JW*: *Avg*.	19.25 a	19.74 a	19.05 a	23.45 a
*JW*, *↓ or ↑ (%)*		2.5	-1.0	21.8
*MW*, *C*.*V*.	7.1	14.5	13.0	24.4
*MW*, *Avg*.	18.18 a	17.81 a	17.85 a	21.95 b
*MW*, *↓or ↑ (%)*		-2.0	-1.8	20.7
*↓ (%)*	-5.6 ^NS^	-9.8 ^NS^	-6.3 ^NS^	-6.4 ^NS^
Holocellul. (%)				
*JW*, *C*.*V*.	5.8	5.3	8.4	7.3
*JW*: *Avg*.	73.55 a	72.28 a	68.84 a	61.57 b
*JW*, *↓ (%)*		-1.7	-6.4	-16.3
*MW*, *C*.*V*.	6.9	3.9	3.8	6.5
*MW*, *Avg*.	73.89 a	74.34 a	74.60 a	68.86 b
*MW*, *↓or ↑ (%)*		0.6	1.0	-6.8
*↑ (%)*	0.5 *	2.9 *	8.4 *	11.8 *
Arabinose (%)				
*JW*, *C*.*V*.	8.7	27.8	6.2	68.2
*JW*: *Avg*.	0.23 a	0.15 b	0.12 bc	0.09 c
*JW*, *↓ (%)*		-34.8	-47.8	-60.9
*MW*, *C*.*V*.	20.7	21.1	29.0	31.5
*MW*, *Avg*.	0.19 a	0.16 b	0.12 bc	0.09 c
*MW*, *↑ (%)*		-15.8	-36.8	-52.6
*↓ or ↑ (%)*	-17.4 ^NS^	6.7 ^NS^	0.0 ^NS^	0.0 ^NS^
Galactose (%)				
*JW*, *C*.*V*.	14.2	58.3	137.8	
*JW*: *Avg*.	0.19 a	0.11 ab	0.04 bc	0.00 c
*JW*, *↓ (%)*		-42.1	-78.9	-100.0
*MW*, *C*.*V*.	34.3	97.2	145.9	
*MW*, *Avg*.	0.19 a	0.10 ab	0.06 bc	0.00 c
*MW*, *↓ (%)*		-47.4	-68.4	-100.0
*↓ or ↑ (%)*	0.0 ^NS^	-9.1 ^NS^	50.0 ^NS^	0.0 ^NS^
Xylose (%)				
*JW*, *C*.*V*.	8.7	22.6	10.9	31.6
*JW*: *Avg*.	13.45 a	12.34 a	11.55 ab	8.78 b
*JW*, *↓ (%)*		-8.3	-14.1	-34.7
*MW*, *C*.*V*.	16.5	17.8	15.3	17.2
*MW*, *Avg*.	11.51 a	11.65 a	11.13 ab	9.73 b
*MW*, *↓or ↑ (%)*		1.2	-3.3	-15.5
*↓ or ↑ (%)*	-14.4 ^NS^	-5.6 ^NS^	-3.6 ^NS^	10.8 ^NS^
Mannose (%)				
*JW*, *C*.*V*.				
*JW*: *Avg*.	ND	ND	ND	ND
*JW*, *↓ or ↑ (%)*				
*MW*, *C*.*V*.				
*MW*, *Avg*.	ND	ND	ND	ND
*MW*, *↓or ↑ (%)*				
*↓ or ↑ (%)*				
Glucose (%)				
*JW*, *C*.*V*.	8.2	9.5	11.3	5.2
*JW*: *Avg*.	59.69 a	59.68 a	57.12 a	52.10 a
*JW*, *↓ or ↑ (%)*		0.0	-4.3	-11.7
*MW*, *C*.*V*.	9.6	7.9	6.5	6.3
*MW*, *Avg*.	62.00 a	62.44 a	63.28 a	59.04 a
*MW*, *↓or ↑ (%)*		0.7	2.1	-4.8
*↑ (%)*	3.9 *	4.6 *	10.8 *	12.0 *

Where: JW—juvenile wood; MW—mature wood; C.V.—coefficient of variation; Avg.—average; ND—non-detected; ↑—increase; ↓—reduction; different letters—significant difference by Scheirer-Ray-Hare and Tukey tests at 5% significance between thermal treatment, to galactose and all others variables, respectively

*—significant difference by “F” test at 5% significance between juvenile and mature woods; same letters and ^NS^, non-significant difference.

Thermal treatment caused significant increase (*P*<0.001) in extractive content for juvenile and mature wood (up to 98.7% and 63.8% respectively) and a significant decrease (*P*<0.001) in holocellulose content (as much as 16.3% for juvenile wood and 6.8% for mature wood) (Table 1). The greatest effect for both juvenile and mature wood occurred at 220°C. It was assumed that the increase in extractive content during thermal treatment was due to a degradation of holocellulose.

Several other studies have shown that heat treatment of wood leads to hemicellulose degradations, resulting in an increase of the extractives and lignin contents [8, 9]. A significant increase (*P* = 0.012) in lignin content for both juvenile (21.8%) and mature wood (20.7%) occurred with the treatment at 220°C (Table 1). These results are similar to those reported by Brito et al [9], and Severo et al [8]. Brito et al [9] reported that as a general trend, the relative mass proportion of lignin increased with both elevated temperature and extended treatment time, with a simultaneous decrease in sugar content.

The interaction between type of wood and the treatment is not significant to extractive (P = 0.343), lignin (P = 0.990), holocellulose (P = 0.267), arabinose (P = 0.889), xylose (P = 0.466), mannose (P = 1.000) and glucose (P = 0.703) contents. However, the influence of thermal treatment in the magnitude of chemical properties of mature wood was lower than in juvenile wood. Previous studies about the chemical properties of untreated rubber wood can help explain this behavior. According to Kadir and Sudin [26] this species presents high fructose, glucose, sucrose, maltose and starch contents in cellular lumen. However, these compounds are relatively low at the central part of the stem (regions where occurs the juvenile wood) and great in the periphery (regions where occurs the mature wood). Moreover, according to explained by Shanks and Gunaratne [27] heat treatments promotes the gelatinization of starch, which results in a lower heat capacity. Thus, these sugars and starches, which are present only in the cellular lumen of periphery of the *H*. *brasiliensis* stem, can delay the thermal degradation of wood cell wall and, therefore, maintain the mature wood closer to its original condition. Thus, kinetic events related to heat capacity should be studied in future studies with thermally-modified wood that contains high starch content.

Thermal treatment caused significant decrease in arabinose (*P*<0.001), galactose (*P*<0.001), and xylose (*P* = 0.006) contents of up to 60.9%, 100% and 34.7%, in juvenile wood, while in mature wood decrease of as much as 52.6%, 100%, and 15.5%, respectively. Mannose wasn’t found. These compounds are responsible for hemicellulose formation [8–10]. There was no significant difference (*P* = 1.000) between juvenile and mature wood.

No change was observed in the glucose content. The lack of change in glucose content may occur since as the hemicelluloses degrade there is an increase in the degree of crystallinity and the width of the crystalalites of cellulose and the amorphous region of cellulose [6, 11–15, 28].

### Equilibrium moisture contents

The p value of Kolmogorov-Smirnov’s normality test for equilibrium moisture contents of untreated wood before exposure to fungus *P*. *sanguineus* and *G*. *trabeum* presented respective p value of 0.592 and 0.620. The EMCs of untreated wood (Table 2) were between 8.56% and 9.0% for juvenile and mature wood before exposure to fungus, when acclimatized at 21°C and 65% RH. The EMC of untreated wood was lower than usual due to the phenomenon known as *hysteresis*. The greatest reduction in EMC occurred at for wood treated at 220°C. According to Bhuiyan et al [11], Vernois [12], Waskett and Selmes [13], Wikberg and Maunu [14], Metsä-Kortelainen et al [15], Bächle et al [28], Calonego et al [6] and Esteves et al [10], heating of wood at high temperatures causes degradation of the hemicelluloses and the amorphous region of cellulose, thereby contributing to the increase in the degree of crystallinity of this polymer. According to these authors, cross linkage between lignin and polymers occurs owing to the thermal degradation of the wood, which is responsible for the decrease in the hygroscopicity and improvement of the dimensional stability.

**Table 2:** Effect of thermal modification on the biological durability of juvenile and mature woods from *Hevea brasiliensis* at fungi *Picnoporus sanguineous* and *Gloeophyllum trabeum*.

Properties	Treatment temperature			
	Untreated	180°C	200°C	220°C
*P*. *sanguineus* test				
Initial EMC (%)				
*JW*, *C*.*V*.	4.0	5.9	8.3	11.1
*JW*: *Avg*.	8.56 a	7.93 b	7.35 b	6.27 c
*JW*, *↓ (%)*		-7.4	-14.1	-26.8
*MW*, *C*.*V*.	3.9	5.6	6.0	11.0
*MW*, *Avg*.	9.00 a	7.82 b	7.75 b	6.35 c
*MW*, *↓ (%)*		-13.1	-13.9	-29.4
*↓ or ↑ (%)*	5.1 *	-1.4 *	5.4 *	1.3 *
Weight loss (%)				
*JW*, *C*.*V*.	20.9	23.1	26.9	24.5
*JW*: *Avg*.	37.24 a	31.15 ab	27.80 b	23.70 b
*JW*, *↓ (%)*		-16.4	-25.3	-36.4
*MW*, *C*.*V*.	14.9	12.9	17.4	21.7
*MW*, *Avg*.	40.78 a	41.43 ab	39.58 b	35.84 b
*MW*, *↓or ↑ (%)*		1.6	-2.9	-12.1
*↑ (%)*	9.5 *	33.0 *	42.4 *	51.2 *
*G*. *trabeum* test				
Initial EMC (%)				
*JW*, *C*.*V*.	4.5	6.2	6.5	16.0
*JW*: *Avg*.	8.90 a	7.60 b	7.47 b	6.12 c
*JW*, *↓ (%)*		-14.6	-16.1	-31.2
*MW*, *C*.*V*.	4.6	6.8	4.5	6.3
*MW*, *Avg*.	8.92 a	8.11 b	7.80 b	6.13 c
*MW*, *↓ (%)*		-9.1	-12.6	-31.3
*↑ (%)*	0.2 *	6.7 *	4.4 *	0.2 *
Weight loss (%)				
*JW*, *C*.*V*.	32.9	60.2	50.3	53.1
*JW*: *Avg*.	29.26 a	19.79 b	13.83 bc	9.21 c
*JW*, *↓ (%)*		-32.4	-52.7	-68.5
*MW*, *C*.*V*.	34.1	69.4	29.2	60.5
*MW*, *Avg*.	27.56 a	25.65 a	25.01 a	11.36 b
*MW*, *↓ (%)*		-6.9	-9.3	-58.8
*↓ or ↑ (%)*	-5.8 ^NS^	29.6 ^NS^	80.8 *	23.3 ^NS^

Where: EMC—equilibrium moisture content; JW—juvenile wood; MW—mature wood; C.V.—coefficient of variation; Avg.—average; ND—non-detected; ↑—increase; ↓—reduction; different letters—significant difference by Tukey test at 5% significance between thermal treatment

*—significant difference by “F” test at 5% significance between juvenile and mature woods; same letters and ^NS^—non-significant difference.

The EMC in juvenile wood was significantly lower than that for mature wood, both for white-rot (*P*<0.001) and brown-rot (*P* = 0.030) fungi. Similar results were obtained by Lenth and Kamke [29] in a study with *Populus tremuloides*, *Pinus taeda* and *Liriodendron tulipifera*. However, the interaction between type of wood and the treatment is not significant to EMC for white-rot (P = 0.131) and brown-rot (P = 0.205) fungi.

### Decay resistance from thermally modified wood

The p value of Kolmogorov-Smirnov’s normality test for weight loss of wood exposure to fungus *P*. *sanguineus* and *G*. *trabeum* presented respective p value of 0.667 and 0.305. The weight loss was 37.24% and 40.78% for untreated juvenile and mature wood samples (Table 2) incubated with the white-rot fungus (*P*. *sanguineus)*, and 29.26% and 27.56% for juvenile and mature wood samples incubated with the brown-rot fungus *(G*. *trabeum)*. Based on these results, *H*. *brasiliensis* wood can be classified according to ASTM D-2017 [23] in the class of moderatel resistant to decay, one in which the weight loss due to decay varies between 25% and 44%.

Thermally modified juvenile wood incubated with the white-rot and brown-rot showed reductions of up to 36.4% and 68.5% in the weight losses when compared with the untreated wood. Thermally modified mature wood incubated with the same fungi presented reductions of up to 12.1% and 58.8% in these respective weight losses (Table 2).

As expected, there was a reduction of weight loss at higher temperatures, as corroborated by the significant differences (*P*<0.001) among treatments. The greatest wood decay resistance occurred at 220°C, both for juvenile and mature wood. These results are similar to those reported by Waskett and Selmes [13], Metsä-Kortelainen et al [15] and Calonego et al [6], who showed that an increase in the length of time and temperature during thermal treatment increased the decay resistance of Pine wood, *Pinus sylvestris* and *Picea abies*, and *Eucalyptus grandis* wood, respectively.

The decrease in weight loss caused by decay fungi (Table 2) has already been explained by several authors, among them Weiland & Guyonnet [4], Hakkou et al [5], Calonego et al [6] and Severo et al [8]. According to these authors there were changes in the chemical composition of wood, mainly the unavailability of food (hemicelluloses) to the fungi, the production of new molecules that act as fungicides, reduction in moisture content, and the cross-linking between lignin and the polymer from the thermally degraded cellulose.

This study also showed that mature wood was significantly more susceptible to degradation than the juvenile wood for both brown rot and white rot (Table 2). This can be partially explained by the chemical analyses (Table 1) which shows that the extractive content was significantly larger in juvenile wood than in mature wood, both untreated and thermally-modified wood. Several studies have associated low wood resistance to decay as a result of a low concentration of extractives since extractives may act as a fungicide [30–32].

However, the interaction between type of wood and the treatment is not significant to weight loss for white-rot (P = 0.058) and is significant to brown-rot (P = 0.039) fungi.

Most importantly, heat treatment increased *H*. *brasiliensis* wood resistance fungi degradation according to ASTM D-2017 [23]. However, this effect appears to be more effective in wood incubated with brown-rot fungus than with white rot fungi (Table 2). Thermally modifying juvenile wood at 180°C, 200°C and 220°C and mature wood at 220°C results in changing the decay resistance classification from moderately resistant to resistant, one in which the weight loss due to decay varies between 11% and 24%. However, wood incubated with white rot fungi only showed change its fungal resistance at the treatment of 220°C.

## Conclusions

It was concluded that the thermal modification caused: (1) a significant decrease in the EMC; (2) a decrease in holocelluloses, arabinose, galactose and xylose content, no change in glucose content; a significant increase in the extractive content, and a proportional increase in the lignin content at 220°C; and (3) a significant increase in wood decay resistance against both *Pycnoporus sanguineus* and *Gloeophyllum trabeum* decay fungi. A treatment at 220°C resulted in a change in ASTM D-2017 [23] wood decay resistance class from moderately resistant to resistant. The influence of thermal treatment on the magnitude of chemical properties and decay resistance in mature wood was lower than in juvenile wood.

## Supporting Information

S1 Table Minimal data set of chemical properties of juvenile and mature woods from thermally-modified rubberwood. (DOC)

S2 Table Minimal data set of decay of juvenile and mature woods from thermally-modified rubberwood at fungus *Pycnoporus sanguineus*. (DOC)

S3 Table Minimal data set of decay of juvenile and mature woods from thermally-modified rubberwood at fungus *Gloeophylum trabeum*. (DOC)
