# Supplementary material for: Changes in the Chemical Composition and Decay Resistance of Thermally-Modified Hevea brasiliensis Wood
Source: PLoS One. 2016 Mar 17;11(3):e0151353. doi: 10.1371/journal.pone.0151353 (PMC4795606; doi:10.1371/journal.pone.0151353)
Supplement: S1 Table — (DOC) [file pone.0151353.s001.doc]

S1 Table. Minimal data set of chemical properties of juvenile and mature woods from thermally-modified rubberwood.

| Type of Wood | Treatment | N | Extractives % | Klason Lignin % | Holocelluloses % | Arabinose % | Galactose % | Xylose % | Mannose % | Glucose % |
| --- | --- | --- | --- | --- | --- | --- | --- | --- | --- | --- |
| 1 | 1 | 1 | 5.23 | 17.03 | 76.71 | 0.25 | 0.2 | 11.86 | 0 | 64.41 |
| 1 | 1 | 2 | 7.25 | 21.47 | 66.59 | 0.23 | 0.2 | 14.28 | 0 | 51.87 |
| 1 | 1 | 3 | 5.91 | 17.43 | 74.76 | 0.19 | 0.16 | 12.53 | 0 | 61.88 |
| 1 | 1 | 4 | 5.51 | 20.13 | 77.01 | 0.24 | 0.22 | 14.4 | 0 | 62.15 |
| 1 | 1 | 5 | 5.9 | 20.17 | 72.68 | 0.23 | 0.17 | 14.16 | 0 | 58.13 |
| 1 | 2 | 1 | 5.95 | 20.1 | 73.49 | 0.08 | 0 | 7.53 | 0 | 65.88 |
| 1 | 2 | 2 | 12.65 | 21.63 | 66.06 | 0.19 | 0.17 | 14.65 | 0 | 51.05 |
| 1 | 2 | 3 | 6 | 17.33 | 76.61 | 0.15 | 0.11 | 12.95 | 0 | 63.39 |
| 1 | 2 | 4 | 8.47 | 20.23 | 73.13 | 0.16 | 0.13 | 13.77 | 0 | 59.07 |
| 1 | 2 | 5 | 9.47 | 19.43 | 72.11 | 0.15 | 0.14 | 12.82 | 0 | 58.99 |
| 1 | 3 | 1 | 8.45 | 18.47 | 74.18 | 0.12 | 0 | 9.47 | 0 | 64.6 |
| 1 | 3 | 2 | 10.85 | 21.13 | 60.6 | 0.13 | 0.12 | 12.43 | 0 | 47.93 |
| 1 | 3 | 3 | 13.08 | 18.23 | 69.16 | 0.13 | 0 | 11.85 | 0 | 57.19 |
| 1 | 3 | 4 | 8.93 | 17.73 | 74.33 | 0.13 | 0.1 | 12.61 | 0 | 61.49 |
| 1 | 3 | 5 | 12.98 | 19.7 | 65.95 | 0.11 | 0 | 11.42 | 0 | 54.42 |
| 1 | 4 | 1 | 10.5 | 18.87 | 67.73 | 0.19 | 0 | 12.79 | 0 | 54.75 |
| 1 | 4 | 2 | 14.34 | 26.57 | 59.37 | 0.06 | 0 | 7.38 | 0 | 51.93 |
| 1 | 4 | 3 | 9.35 | 17.43 | 64.2 | 0.06 | 0 | 7.76 | 0 | 56.37 |
| 1 | 4 | 4 | 13.99 | 24.73 | 60.38 | 0.07 | 0 | 10.28 | 0 | 50.03 |
| 1 | 4 | 5 | 11.01 | 29.67 | 56.19 | 0.04 | 0 | 5.71 | 0 | 50.43 |
| 2 | 1 | 1 | 5.91 | 16.97 | 66.44 | 0.15 | 0.11 | 10.65 | 0 | 55.54 |
| 2 | 1 | 2 | 5.86 | 19.93 | 71.09 | 0.23 | 0.28 | 13.39 | 0 | 57.18 |
| 2 | 1 | 3 | 5.85 | 16.87 | 79.32 | 0.16 | 0.16 | 8.75 | 0 | 70.26 |
| 2 | 1 | 4 | 5.55 | 18.83 | 76.71 | 0.19 | 0.21 | 11.7 | 0 | 64.61 |
| 2 | 1 | 5 | 5.99 | 18.3 | 75.9 | 0.23 | 0.2 | 13.07 | 0 | 62.4 |
| 2 | 2 | 1 | 6.03 | 16.7 | 75.63 | 0.14 | 0 | 10.94 | 0 | 64.54 |
| 2 | 2 | 2 | 7.69 | 20.87 | 70.4 | 0.2 | 0.21 | 13.75 | 0 | 56.24 |
| 2 | 2 | 3 | 5.9 | 14.97 | 77.35 | 0.12 | 0 | 8.42 | 0 | 68.8 |
| 2 | 2 | 4 | 13.57 | 16.33 | 76.14 | 0.14 | 0.14 | 12.2 | 0 | 63.67 |
| 2 | 2 | 5 | 7.99 | 20.2 | 72.19 | 0.18 | 0.13 | 12.91 | 0 | 58.96 |
| 2 | 3 | 1 | 6.45 | 15.23 | 77.31 | 0.07 | 0 | 8.67 | 0 | 68.57 |
| 2 | 3 | 2 | 7.98 | 21.2 | 73.74 | 0.17 | 0.18 | 13.12 | 0 | 60.28 |
| 2 | 3 | 3 | 7.53 | 16.7 | 74.84 | 0.12 | 0 | 10.28 | 0 | 64.44 |
| 2 | 3 | 4 | 7.69 | 17.03 | 76.83 | 0.13 | 0 | 11.66 | 0 | 65.04 |
| 2 | 3 | 5 | 10.67 | 19.07 | 70.26 | 0.13 | 0.1 | 11.94 | 0 | 58.09 |
| 2 | 4 | 1 | 10.12 | 17.97 | 69.37 | 0.12 | 0 | 12.11 | 0 | 57.14 |
| 2 | 4 | 2 | 9.87 | 24.83 | 61.4 | 0.07 | 0 | 7.74 | 0 | 53.6 |
| 2 | 4 | 3 | 7.53 | 17.87 | 73.31 | 0.1 | 0 | 10.23 | 0 | 62.97 |
| 2 | 4 | 4 | 8.71 | 19.03 | 70.45 | 0.07 | 0 | 9.99 | 0 | 60.38 |
| 2 | 4 | 5 | 11.52 | 30.03 | 69.75 | 0.06 | 0 | 8.58 | 0 | 61.11 |

where: N - number of replication; Type of wood 1 - Juvenile Wood, 2 - Mature Wood; Treatment 1 - Untreated, 2 - 180ºC, 3 - 200ºC, and 3 - 220ºC.
