# Supplementary material for: Changes in the Chemical Composition and Decay Resistance of Thermally-Modified Hevea brasiliensis Wood
Source: PLoS One. 2016 Mar 17;11(3):e0151353. doi: 10.1371/journal.pone.0151353 (PMC4795606; doi:10.1371/journal.pone.0151353)
Supplement: S2 Table — (DOC) [file pone.0151353.s002.doc]

S2 Table. Minimal data set of decay of juvenile and mature woods from thermally-modified rubberwood at fungus *Pycnoporus sanguineus*.

| Type of Wood | Treatment | N | Initial EMC % | WL % |
| --- | --- | --- | --- | --- |
| 1 | 1 | 1 | 8.466 | 28.3 |
| 1 | 1 | 2 | 8.443 | 36.4 |
| 1 | 1 | 3 | 8.201 | 34.4 |
| 1 | 1 | 4 | 9.164 | 37.2 |
| 1 | 1 | 5 | 8.556 | 40.9 |
| 1 | 1 | 6 | 8.824 | 42.5 |
| 1 | 1 | 7 | 8.602 | 31.5 |
| 1 | 1 | 8 | 8.378 | 28.9 |
| 1 | 1 | 9 | 8.056 | 27.8 |
| 1 | 1 | 10 | 8.333 | 54.7 |
| 1 | 1 | 11 | 8.646 | 47.8 |
| 1 | 1 | 12 | 8.883 | 45.6 |
| 1 | 1 | 13 | 9.186 | 37 |
| 1 | 1 | 14 | 8.437 | 32.8 |
| 1 | 1 | 15 | 8.158 | 32.9 |
| 1 | 2 | 1 | 7.246 | 23.2 |
| 1 | 2 | 2 | 7.949 | 25.9 |
| 1 | 2 | 3 | 8.333 | 25.3 |
| 1 | 2 | 4 | 8.179 | 34.6 |
| 1 | 2 | 5 | 8.134 | 38.8 |
| 1 | 2 | 6 | 8.684 | 33.4 |
| 1 | 2 | 7 | 8.128 | 24.6 |
| 1 | 2 | 8 | 8.179 | 25.9 |
| 1 | 2 | 9 | 8.108 | 23.8 |
| 1 | 2 | 10 | 8.136 | 45.4 |
| 1 | 2 | 11 | 8.406 | 42.9 |
| 1 | 2 | 12 | 7.521 | 26.2 |
| 1 | 2 | 13 | 7.102 | 34.4 |
| 1 | 2 | 14 | 7.459 | 28.2 |
| 1 | 2 | 15 | 7.429 | 34.9 |
| 1 | 3 | 1 | 6.015 | 19.3 |
| 1 | 3 | 2 | 7.105 | 21.1 |
| 1 | 3 | 3 | 7.219 | 19.8 |
| 1 | 3 | 4 | 7.796 | 35.5 |
| 1 | 3 | 5 | 7.657 | 28.1 |
| 1 | 3 | 6 | 7.958 | 35 |
| 1 | 3 | 7 | 7.371 | 23.6 |
| 1 | 3 | 8 | 7.33 | 22.3 |
| 1 | 3 | 9 | 7.673 | 24 |
| 1 | 3 | 10 | 8 | 38.9 |
| 1 | 3 | 11 | 7.778 | 34.4 |
| 1 | 3 | 12 | 7.859 | 41.5 |
| 1 | 3 | 13 | 6.045 | 22.9 |
| 1 | 3 | 14 | 7.087 | 29.4 |
| 1 | 3 | 15 | 7.365 | 21.2 |
| 1 | 4 | 1 | 6.753 | 24.7 |
| 1 | 4 | 2 | 6.842 | 21.8 |
| 1 | 4 | 3 | 6.361 | 21.9 |
| 1 | 4 | 4 | 5.556 | 23.3 |
| 1 | 4 | 5 | 5.714 | 23.1 |
| 1 | 4 | 6 | 5.6 | 21.9 |
| 1 | 4 | 7 | 7.16 | 24.1 |
| 1 | 4 | 8 | 7.614 | 24.4 |
| 1 | 4 | 9 | 7.294 | 20 |
| 1 | 4 | 10 | 6.011 | 34.4 |
| 1 | 4 | 11 | 6 | 36.3 |
| 1 | 4 | 12 | 6.105 | 29.4 |
| 1 | 4 | 13 | 5.572 | 17 |
| 1 | 4 | 14 | 5.932 | 17.8 |
| 1 | 4 | 15 | 5.539 | 15.7 |
| 2 | 1 | 1 | 8.4 | 45.55 |
| 2 | 1 | 2 | 9.45 | 44.36 |
| 2 | 1 | 3 | 9.45 | 50.13 |
| 2 | 1 | 4 | 8.92 | 39.04 |
| 2 | 1 | 5 | 9 | 47.81 |
| 2 | 1 | 6 | 9 | 47.5 |
| 2 | 1 | 7 | 8.7 | 33.82 |
| 2 | 1 | 8 | 8.44 | 32.74 |
| 2 | 1 | 9 | 8.86 | 28.44 |
| 2 | 1 | 10 | 9.09 | 41.76 |
| 2 | 1 | 11 | 8.94 | 40.78 |
| 2 | 1 | 12 | 9.09 | 42.51 |
| 2 | 1 | 13 | 9.64 | 36.09 |
| 2 | 1 | 14 | 9.14 | 41.94 |
| 2 | 1 | 15 | 8.83 | 39.22 |
| 2 | 2 | 1 | 8.07 | 43.77 |
| 2 | 2 | 2 | 7.82 | 39.85 |
| 2 | 2 | 3 | 7.23 | 42.64 |
| 2 | 2 | 4 | 8.31 | 43.38 |
| 2 | 2 | 5 | 8.61 | 49.72 |
| 2 | 2 | 6 | 8.33 | 39.66 |
| 2 | 2 | 7 | 7.11 | 37.11 |
| 2 | 2 | 8 | 7.43 | 44.31 |
| 2 | 2 | 9 | 8.05 | 29.87 |
| 2 | 2 | 10 | 7.52 | 40.11 |
| 2 | 2 | 11 | 7.59 | 37.13 |
| 2 | 2 | 12 | 7.86 | 49.32 |
| 2 | 2 | 13 | 7.38 | 45.36 |
| 2 | 2 | 14 | 7.84 | 34.73 |
| 2 | 2 | 15 | 8.15 | 44.57 |
| 2 | 3 | 1 | 6.91 | 29.41 |
| 2 | 3 | 2 | 7.32 | 34.6 |
| 2 | 3 | 3 | 7.91 | 45.8 |
| 2 | 3 | 4 | 8.21 | 44.03 |
| 2 | 3 | 5 | 8.21 | 45.64 |
| 2 | 3 | 6 | 7.84 | 53.92 |
| 2 | 3 | 7 | 7.75 | 42.38 |
| 2 | 3 | 8 | 7.87 | 47.22 |
| 2 | 3 | 9 | 7.29 | 40.45 |
| 2 | 3 | 10 | 7.67 | 34.66 |
| 2 | 3 | 11 | 7.98 | 32.48 |
| 2 | 3 | 12 | 8.75 | 32.65 |
| 2 | 3 | 13 | 7.67 | 32.95 |
| 2 | 3 | 14 | 7.78 | 38.62 |
| 2 | 3 | 15 | 7.1 | 38.92 |
| 2 | 4 | 1 | 6.27 | 36.03 |
| 2 | 4 | 2 | 6.04 | 42.26 |
| 2 | 4 | 3 | 8.42 | 38.12 |
| 2 | 4 | 4 | 6.49 | 29.61 |
| 2 | 4 | 5 | 6.58 | 30.14 |
| 2 | 4 | 6 | 5.96 | 27.2 |
| 2 | 4 | 7 | 6.23 | 40.26 |
| 2 | 4 | 8 | 6.23 | 49.13 |
| 2 | 4 | 9 | 6.9 | 36.07 |
| 2 | 4 | 10 | 6.74 | 42.82 |
| 2 | 4 | 11 | 5.8 | 45.8 |
| 2 | 4 | 12 | 6.25 | 41.19 |
| 2 | 4 | 13 | 5.49 | 26.3 |
| 2 | 4 | 14 | 5.52 | 28.2 |
| 2 | 4 | 15 | 6.25 | 24.43 |

where: N - number of replication; EMC - Equilibrium Moisture Content; WL - Weight Loss; Type of wood 1 - Juvenile Wood, 2 - Mature Wood; Treatment 1 - Untreated, 2 - 180ºC, 3 - 200ºC, and 3 - 220ºC.
