# Supplementary material for: Changes in the Chemical Composition and Decay Resistance of Thermally-Modified Hevea brasiliensis Wood
Source: PLoS One. 2016 Mar 17;11(3):e0151353. doi: 10.1371/journal.pone.0151353 (PMC4795606; doi:10.1371/journal.pone.0151353)
Supplement: S3 Table — (DOC) [file pone.0151353.s003.doc]

S3 Table. Minimal data set of decay of juvenile and mature woods from thermally-modified rubberwood at fungus *Gloeophylum trabeum*.

| Type of Wood | Treatment | N | Initial EMC % | WL % |
| --- | --- | --- | --- | --- |
| 1 | 1 | 1 | 8.586 | 30.8 |
| 1 | 1 | 2 | 9.737 | 21.1 |
| 1 | 1 | 3 | 9.091 | 20.6 |
| 1 | 1 | 4 | 8.639 | 47.6 |
| 1 | 1 | 5 | 9.291 | 16.4 |
| 1 | 1 | 6 | 9.231 | 23.6 |
| 1 | 1 | 7 | 8.672 | 46.6 |
| 1 | 1 | 8 | 8.767 | 24.9 |
| 1 | 1 | 9 | 8.245 | 32.2 |
| 1 | 1 | 10 | 9.341 | 28.6 |
| 1 | 1 | 11 | 9.117 | 37.9 |
| 1 | 1 | 12 | 8.516 | 38.7 |
| 1 | 1 | 13 | 8.901 | 22.8 |
| 1 | 1 | 14 | 8.861 | 21 |
| 1 | 1 | 15 | 8.521 | 26.1 |
| 1 | 2 | 1 | 7.652 | 7.7 |
| 1 | 2 | 2 | 7.031 | 25.3 |
| 1 | 2 | 3 | 7.552 | 20.6 |
| 1 | 2 | 4 | 7.914 | 8.4 |
| 1 | 2 | 5 | 8.505 | 25.8 |
| 1 | 2 | 6 | 7.756 | 25.2 |
| 1 | 2 | 7 | 7.754 | 8 |
| 1 | 2 | 8 | 7.653 | 15.1 |
| 1 | 2 | 9 | 7.349 | 12.9 |
| 1 | 2 | 10 | 8.021 | 25.1 |
| 1 | 2 | 11 | 7.821 | 46.1 |
| 1 | 2 | 12 | 8.108 | 41.6 |
| 1 | 2 | 13 | 7.102 | 9.1 |
| 1 | 2 | 14 | 6.793 | 13.6 |
| 1 | 2 | 15 | 6.997 | 12.5 |
| 1 | 3 | 1 | 6.849 | 16.7 |
| 1 | 3 | 2 | 6.868 | 11.5 |
| 1 | 3 | 3 | 7.967 | 4.1 |
| 1 | 3 | 4 | 7.617 | 22.6 |
| 1 | 3 | 5 | 7.013 | 13.5 |
| 1 | 3 | 6 | 7.046 | 6 |
| 1 | 3 | 7 | 7.937 | 26.7 |
| 1 | 3 | 8 | 7.98 | 10 |
| 1 | 3 | 9 | 7.592 | 13.4 |
| 1 | 3 | 10 | 8.264 | 19.3 |
| 1 | 3 | 11 | 7.714 | 22.6 |
| 1 | 3 | 12 | 7.778 | 17.5 |
| 1 | 3 | 13 | 6.736 | 11.7 |
| 1 | 3 | 14 | 7.222 | 5.8 |
| 1 | 3 | 15 | 7.438 | 6.1 |
| 1 | 4 | 1 | 6.648 | 22.4 |
| 1 | 4 | 2 | 6.944 | 5.8 |
| 1 | 4 | 3 | 6.775 | 10.3 |
| 1 | 4 | 4 | 4.604 | 6.1 |
| 1 | 4 | 5 | 4.8 | 6.7 |
| 1 | 4 | 6 | 4.336 | 7 |
| 1 | 4 | 7 | 7.039 | 13.6 |
| 1 | 4 | 8 | 6.872 | 6.2 |
| 1 | 4 | 9 | 6.923 | 3.8 |
| 1 | 4 | 10 | 6.609 | 12.6 |
| 1 | 4 | 11 | 6.647 | 13.6 |
| 1 | 4 | 12 | 7.102 | 11.9 |
| 1 | 4 | 13 | 5.294 | 6.5 |
| 1 | 4 | 14 | 5.602 | 5.9 |
| 1 | 4 | 15 | 5.605 | 5.6 |
| 2 | 1 | 1 | 9.05 | 39.36 |
| 2 | 1 | 2 | 8.14 | 33.84 |
| 2 | 1 | 3 | 8.75 | 20.95 |
| 2 | 1 | 4 | 9.37 | 35.81 |
| 2 | 1 | 5 | 8.89 | 28.3 |
| 2 | 1 | 6 | 8.42 | 20.66 |
| 2 | 1 | 7 | 8.43 | 21.31 |
| 2 | 1 | 8 | 8.6 | 15.16 |
| 2 | 1 | 9 | 8.77 | 24.17 |
| 2 | 1 | 10 | 8.78 | 16.49 |
| 2 | 1 | 11 | 9.33 | 18.39 |
| 2 | 1 | 12 | 9.14 | 36.56 |
| 2 | 1 | 13 | 9.31 | 36.7 |
| 2 | 1 | 14 | 9.43 | 21.29 |
| 2 | 1 | 15 | 9.37 | 44.35 |
| 2 | 2 | 1 | 8.42 | 8.42 |
| 2 | 2 | 2 | 8.25 | 44.75 |
| 2 | 2 | 3 | 7.65 | 52.59 |
| 2 | 2 | 4 | 9.37 | 11.85 |
| 2 | 2 | 5 | 8.67 | 12.2 |
| 2 | 2 | 6 | 8.8 | 48.39 |
| 2 | 2 | 7 | 7.62 | 7.62 |
| 2 | 2 | 8 | 7.53 | 6.75 |
| 2 | 2 | 9 | 7.58 | 10.86 |
| 2 | 2 | 10 | 8.03 | 29.09 |
| 2 | 2 | 11 | 8.06 | 14.52 |
| 2 | 2 | 12 | 8.33 | 45.56 |
| 2 | 2 | 13 | 7.9 | 30.52 |
| 2 | 2 | 14 | 8.03 | 48.75 |
| 2 | 2 | 15 | 7.33 | 12.83 |
| 2 | 3 | 1 | 7.51 | 32.17 |
| 2 | 3 | 2 | 8.11 | 16.22 |
| 2 | 3 | 3 | 8.06 | 33.6 |
| 2 | 3 | 4 | 8.02 | 31.83 |
| 2 | 3 | 5 | 8.42 | 28.71 |
| 2 | 3 | 6 | 7.61 | 26.25 |
| 2 | 3 | 7 | 7.48 | 29.91 |
| 2 | 3 | 8 | 7.44 | 26.05 |
| 2 | 3 | 9 | 8.1 | 18.81 |
| 2 | 3 | 10 | 8.09 | 15.61 |
| 2 | 3 | 11 | 8.05 | 33.33 |
| 2 | 3 | 12 | 7.07 | 22.25 |
| 2 | 3 | 13 | 7.71 | 29.14 |
| 2 | 3 | 14 | 7.71 | 21.14 |
| 2 | 3 | 15 | 7.67 | 10.14 |
| 2 | 4 | 1 | 6.7 | 11.26 |
| 2 | 4 | 2 | 6.17 | 31.37 |
| 2 | 4 | 3 | 6.13 | 9.87 |
| 2 | 4 | 4 | 5.28 | 8.97 |
| 2 | 4 | 5 | 5.88 | 8.56 |
| 2 | 4 | 6 | 6.5 | 7.05 |
| 2 | 4 | 7 | 6.23 | 5.99 |
| 2 | 4 | 8 | 6.32 | 7.11 |
| 2 | 4 | 9 | 6.12 | 6.12 |
| 2 | 4 | 10 | 5.73 | 22.64 |
| 2 | 4 | 11 | 6.53 | 9.94 |
| 2 | 4 | 12 | 5.81 | 11.05 |
| 2 | 4 | 13 | 6.03 | 13.51 |
| 2 | 4 | 14 | 6.65 | 7.8 |
| 2 | 4 | 15 | 5.85 | 9.19 |

where: N - number of replication; EMC - Equilibrium Moisture Content; WL - Weight Loss; Type of wood 1 - Juvenile Wood, 2 - Mature Wood; Treatment 1 - Untreated, 2 - 180ºC, 3 - 200ºC, and 3 - 220ºC.
